# The Accuracy of Casts Obtained Using Different Impression Techniques and Impression Materials in Combined Parallel and Angulated Implants: An In Vitro Study

**DOI:** 10.7759/cureus.59193

**Published:** 2024-04-28

**Authors:** Menaga V, Babina Chirom, Kangjam Gunadhar, Shamurailatpam Priyadarshini, Rajesh S Nongthombam, Manikandan P

**Affiliations:** 1 Prosthodontics and Crown and Bridge, Thai Moogambigai Dental College and Hospital, Chennai, IND; 2 Prosthodontics and Crown and Bridge, Regional Institute of Medical Sciences, Imphal, Imphal, IND; 3 Conservative Dentistry and Endodontics, Jawaharlal Nehru Institute of Medical Sciences (JNIMS), Imphal, IND; 4 Conservative Dentistry and Endodontics, Regional Institute of Medical Sciences, Imphal, Imphal, IND; 5 Pedodontics, Sri Ramachandra Dental College, Chennai, IND

**Keywords:** open tray technique, impression material, dental implants, closed tray technique, accuracy

## Abstract

Aim

The aim of this in vitro study was to evaluate and compare the accuracy of casts made from two elastomeric impression materials (polyvinyl siloxane (PVS) and vinylsiloxanether (VSE)) using different impression techniques on parallel and angulated implants.

Materials and methods

The reference model was fabricated using auto-polymerizing acrylic resin on which three implant analogs were placed of which two were parallel to each other and the third at 20-degree mesial angulation. A total of 60 impressions were made of which 30 were by using PVS and 30 by VSE. For each material, 10 impressions were made by closed tray technique, 10 by open tray technique and 10 by open tray with sandblasting and adhesive coating of the impression copings technique. The inter-analog distances of the casts obtained were evaluated and compared with the reference model by a vision measuring machine. Data were analyzed using analysis of variance (ANOVA), Tukey’s Honest Significant Difference (HSD) post hoc and independent samples t-test.

Results

When the inter-analog distances of the duplicate casts were compared with the reference model, the mean error rates for parallel implants decreased in the order of closed tray technique, open tray technique and open tray with sandblasting and adhesive coating of the impression copings technique for both PVS and VSE impression materials. Similarly, the same order was observed for angulated implants for both impression materials. Using the closed tray technique, there was no statistically significant difference in the accuracy of the cast between the two materials for parallel implants (P = 0.525) and also no significant difference between the two materials for angulated implants (P = 0.307). Similarly, there was no statistically significant difference in the accuracy of the cast between the two materials for parallel implants (P = 0.455) and also no significant difference between the two materials for angulated implants (P = 0.519) using the open tray technique. Whereas for the open tray with sandblasting and adhesive coating of the impression copings technique, VSE produced a more accurate cast than PVS for parallel implants and was statistically significant (P = 0.033); however, there was no significant difference between the two materials for angulated implants (P = 0.375).

Conclusion

For parallel implants, VSE by an open tray with sandblasting and adhesive coating of the impression copings technique produced a more accurate cast than PVS. For angulated implants, there was no significant difference between the two materials and it was only the technique that significantly affected the accuracy of the cast.

## Introduction

Dental implants have changed the face of dentistry over the last many years giving a wide variety of predictable and successful treatments for fixed and removable prostheses. Although, Brånemark recommended to place implant in a fairly upright position but there is often a need for placement of angulated implants in clinical situations like severely resorbed ridge and where there are anatomic limitations like close proximity to sinus, nerve and blood vessels in order to enhance the primary stability of implant as it increases implant-to-bone contact area [1-3]. Placing angulated implants avoid the need for a more complex treatment procedure like sinus lift or bone augmentation in many clinical situations [1]. Angulated implants pose a greater risk of inaccuracy in impression than parallel implants and thereby affect the accuracy of the cast [4]. The more the angulation of implants, the more the inaccuracy in impression due to the increased amount of stress generated in the material during the removal of impression [5].

Impression making is the most important clinical step to record accurately the three-dimensional relationships of implant and adjacent structures. An accurate impression is the first step in achieving a precise and passive-fitting prosthesis which is one of the prerequisites for the long-term success of the prosthesis [6,7]. Inaccuracies in impression lead to the fabrication of prostheses with a lack of precision and fit which can further lead to mechanical and/or biological complications [8,9]. Mechanical complications may comprise of loosening, bending and fracture of the implant or prosthetic parts. Biological complications include peri-implantitis, peri-implant mucositis and implant loss due to infection and inflammation [7,10-13].

Factors that affect the accuracy of impression and implant cast are impression technique, type of impression material, splinting or non-splinting of impression copings, type of splinting material, implant angulation and die material accuracy [5,14,15]. With respect to impression materials, both polyether (PE) and polyvinyl siloxane (PVS) have been addressed to be suitable for implant impression. The accuracy and dimensional stability of PVS and PE are well documented [16-19]. In recent years, PVS has become more popular because of its favourable characteristics which include lower modulus of elasticity and higher yield strength compared with PE [20,21]. Recently, a newly formulated elastomeric impression material classified as vinylsiloxanether (VSE) has been commercially available. The composition is intended to incorporate the hydrophilic nature of conventional PE along with the desirable properties of PVS such as elastic recovery and tear resistance. The accuracy of VSE impression material in implant impression is not well documented and needs to be tested.

Most of the studies have evaluated the accuracy of impression by comparing a few individual variables such as impression material, impression technique or implant angulation with very few studies evaluating the effect of all these variables. Moreover, very few studies have evaluated the accuracy of the newly formulated VSE impression material with implant angulation.

Hence, the aim of this in vitro study was to evaluate the accuracy of casts made from two elastomeric impression materials (PVS and VSE) using different impression techniques (open tray, closed tray and closed tray with sandblasting and adhesive coating of the impression copings) on parallel and angulated implants.

## Materials and methods

**Figure 1:**
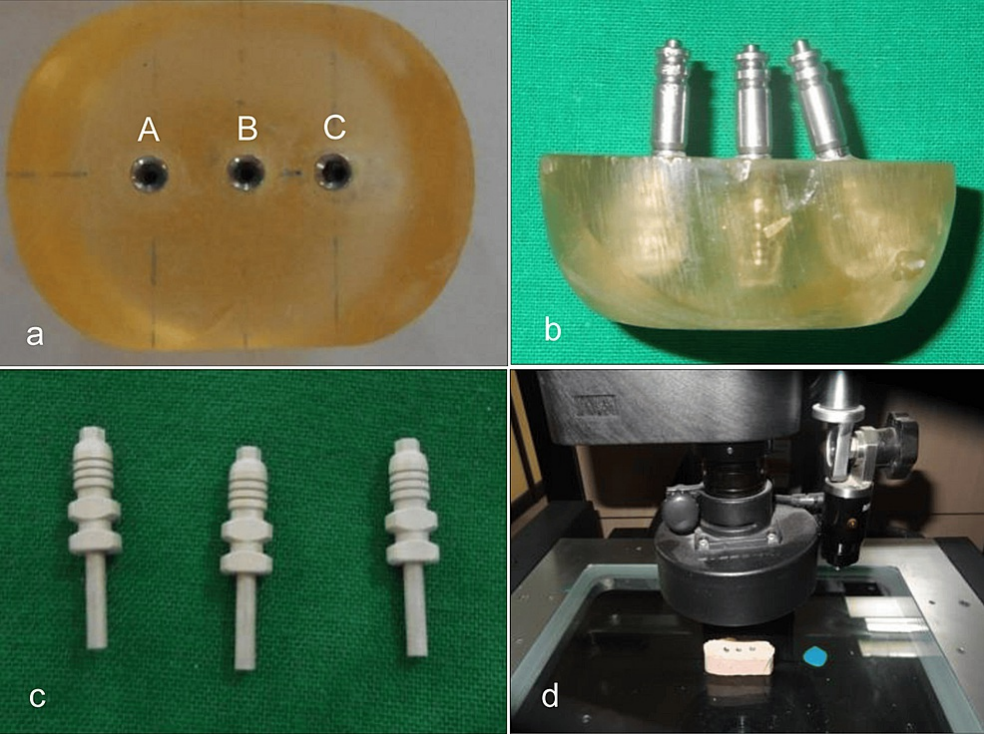
Reference model, impression copings and cast analysis (a) Reference model with implant analogs A, B and C; (b) Reference model with impression copings; (c) Sandblasted impression copings; (d) Cast placed on vision measuring machine.

The reference model was fabricated using auto-polymerizing acrylic resin (DPI® RR Cold Cure, Dental Products of India, Mumbai, India). Three holes for implant analogs (IA, Alpha BioTEC, Israel) were drilled by a milling machine to a depth of 12.5 mm and diameter of 4.2 mm at 10 mm intervals such that the first analog-A and the second analog-B were parallel to each other and perpendicular to the horizontal plane and the third analog-C was placed at 20-degree mesial angulation. The analogs were secured into the holes with auto-polymerizing acrylic resin (Figure 1a). The impression copings were screwed to the IA in the reference model by using a hex driver (Figure 1b).

Sixty custom impression trays (20 closed and 40 open trays) were fabricated using auto-polymerizing acrylic resin. For the fabrication of trays, two sheets of modelling wax (Hindustan Modelling Wax No.2, The Hindustan Dental Products, Hyderabad, India) were adapted over the reference model except at the two cuts located on the sides of the model in order to provide uniform space for impression material and to incorporate tissue stops in the impression trays. Impression was made by using irreversible hydrocolloid (Zhermack Neocolloid, Dentsply India Pvt. Ltd., Bengaluru, India), poured with type IV dental stone (Kalrock, die stone class IV, Kalabhai Karson Pvt. Ltd., Mumbai, India) and the cast thus obtained was used for fabrication of custom trays. The trays for the closed tray technique had retention holes made on the trays but for the open tray technique, there were three windows on the tray for the impression copings. Before impression, the trays were coated with tray adhesive and allowed to dry for 15 minutes. Impressions for both PVS (Flexceed, GC India Dental Pvt. Ltd., Hyderabad, India) and VSE (EXA’lence, GC America, Alsip, USA) were made using the putty-wash technique. Light body impression material was injected around the impression copings and the tray loaded with putty material was seated immediately on the reference model. In the closed tray technique, the custom tray was removed after the polymerization of the impression material. The impression copings were removed from the reference model and attached to IA. The combined units were positioned to the impression. In the open tray technique, the coping screws were unscrewed and the copings were removed along with the impression after the polymerization of the impression material. IA were placed over the impression copings and tightened. Impressions were examined and repeated if any inaccuracies were found.

Initially, 40 impressions were made of which 20 were by using PVS and 20 by VSE. For each material, 10 by closed tray technique and 10 by open tray technique. Then, the impression copings for the open tray technique were modified by sandblasting (Santar Labo 16, Confident Dental Equipments Pvt. Ltd., Bangalore, India) with 50 μm aluminium oxide (Al_2_O_3_) at 2.5 atmospheric pressure to roughen the external surface (Figure 1c). After cleaning in an ultrasonic cleaner, the copings were placed onto the IA placed on the reference model and coping screws were tightened. The impression copings were then coated with tray adhesive and 20 impressions were made by open tray technique of which 10 by using PVS and 10 by VSE.

Hence, a total of 60 impressions were made of which 30 were by using PVS and 30 by VSE. For each material, 10 by closed tray technique, 10 by open tray technique and 10 by open tray with sandblasting and adhesive coating of the impression copings technique. Impressions were boxed and poured using type IV dental stone (Kalrock, die stone class IV, Kalabhai Karson Pvt. Ltd., Mumbai, India) on a vibrator. After the stone sets, the tray was removed and the obtained cast was trimmed.

The top centre of the analog was used for measuring the inter-analog distance. D1 was the distance from the top center of analog-A to the top center of parallelly positioned analog-B and D2 was the distance from the top center of analog-B to the top center of angulated analog-C in the reference model. Similarly, d1 and d2 were distances from analog-A to analog-B and analog-B to analog-C respectively in the duplicate cast.

The reference model and each of the duplicate casts were positioned on the movable table of the vision measuring machine (OPUS Model 3020T, Singapore) (Figure 1d). The readings were recorded and compared to check for the accuracy of all the duplicate casts. The error rate (ER) was calculated by the formula: 

$$ER = \frac{D - d}{D} \times 100\%$$

Statistical analysis was performed using SPSS software for Windows (SPSS, version 18, IBM Corp., Armonk, NY, US). Tukey’s Honest Significant Difference (HSD) post hoc test was used for multiple pairwise comparisons between techniques. One-way analysis of variance (ANOVA) was used to compare the mean error rates between techniques in each material. Independent samples t-test was used to compare mean error rates between materials within the technique. A value of P < 0.05 was considered statistically significant.

## Results

When the inter-analog distances of the duplicate casts were compared with the reference model, Tukey’s HSD post hoc test showed for parallel implants, there was a statistically significant difference between the closed tray technique and the open tray technique (P = 0.011) and also between closed tray technique and open tray with sandblasting and adhesive coating of the impression copings technique (P < 0.001) on the accuracy of cast. Similarly, for angulated implants, there was a statistically significant difference between the closed tray technique and the open tray technique (P = 0.002) and also between the closed tray technique and open tray with sandblasting and adhesive coating of the impression copings technique (P = 0.001). The open tray with sandblasting and adhesive coating of the impression copings technique produced the least error followed by the open tray technique and closed tray technique for both parallel implants and angulated implants (Table 1).

**Table 1:** Tukey’s HSD post hoc test for comparison between techniques *Significant difference (P<0.05). HSD: Honest Significant Difference

Variable	Technique (A)	Technique (B)	Mean difference (A-B)	P
Error rate for parallel implants (%)	Closed tray	Open tray	0.25	0.011*
	Closed tray	Open tray with sandblasting and adhesive coating of impression copings	0.36	<0.001*
	Open tray	Open tray with sandblasting and adhesive coating of impression copings	0.11	0.246
Error rate for angulated implants (%)	Closed tray	Open tray	0.458	0.002*
	Closed tray	Open tray with sandblasting and adhesive coating of impression copings	0.611	0.001*
	Open tray	Open tray with sandblasting and adhesive coating of impression copings	0.153	0.288

One-way ANOVA showed that impressions by using PVS had no statistically significant difference between the techniques on the accuracy of the cast for parallel implants (P = 0.118); however, for angulated implants, there was a significant difference between the techniques on the accuracy of the cast (P < 0.001). Whereas with respect to VSE, there was a statistically significant difference between the techniques on the accuracy of cast for parallel implants (P = 0.003); however, there was no significant difference between the techniques on the accuracy of cast for angulated implants (P = 0.187) (Table 2).

**Table 2:** One-way ANOVA for comparison between techniques in each material *Significant difference (P<0.05). ANOVA: analysis of variance

Material	Variable	Sum of squares		df	Mean square	F	P
Polyvinyl siloxane	Error rate for parallel implants (%)	Between techniques	0.561	2	0.281	2.315	0.118
		Within techniques	3.272	27	0.121		
		Total	3.833	29			
	Error rate for angulated implants (%)	Between techniques	3.825	2	1.912	10.318	<0.001*
		Within techniques	5.005	27	0.185		
		Total	8.830	29			
Vinylsiloxanether	Error rate for parallel implants (%)	Between techniques	0.847	2	0.424	7.142	0.003*
		Within techniques	1.601	27	0.059		
		Total	2.449	29			
	Error rate for angulated implants (%)	Between techniques	0.799	2	0.400	1.783	0.187
		Within techniques	6.050	27	0.224		
		Total	6.849	29			

Impression by using PVS for angulated implants showed that the open tray technique produced a more accurate cast than the closed tray technique with statistical significance (P = 0.006) and also, open tray with sandblasting and adhesive coating of the impression copings technique produced more accurate cast than closed tray technique (P = 0.001). Whereas with respect to impression by using VSE for parallel implants, the open tray with sandblasting and adhesive coating of the impression copings technique produced a more accurate cast than the closed tray technique (P = 0.002) (Table 3).

**Table 3:** Tukey’s HSD post hoc test for multiple comparisons *Significant difference (P<0.05). HSD: Honest Significant Difference

Material	Variable	Technique (A)	Technique (B)	Mean difference (A-B)	P
Polyvinyl siloxane	Error rate for angulated implants (%)	Closed tray	Open tray	0.6551	0.006*
		Closed tray	Open tray with sandblasting and adhesive coating of impression copings	0.8294	0.001*
		Open tray	Open tray with sandblasting and adhesive coating of impression copings	0.1744	0.641
Vinylsiloxanether	Error rate for parallel implants (%)	Closed tray	Open tray	0.2508	0.073
		Closed tray	Open tray with sandblasting and adhesive coating of impression copings	0.4081	0.002*
		Open tray	Open tray with sandblasting and adhesive coating of impression copings	0.1573	0.333

For parallel implants, the open tray with sandblasting and adhesive coating of the impression copings technique produced the most accurate cast followed by the open tray technique and closed tray technique for both PVS and VSE impression materials. The same order was observed for angulated implants for both impression materials. There was no statistically significant difference between the two materials within a technique except in the open tray with sandblasting and adhesive coating of the impression copings technique which revealed that VSE produced a more accurate cast than PVS for parallel implants (P = 0.033) (Table 4).

**Table 4:** Independent samples t-test for comparison between materials within the technique *Significant difference (P<0.05)

Technique	Variable	Material	n	Mean difference	SD	t-test	P
Closed tray	Error rate for parallel implants (%)	Polyvinyl siloxane	10	0.71	0.45	0.648	0.525
		Vinylsiloxanether	10	0.60	0.27		
	Error rate for angulated implants (%)	Polyvinyl siloxane	10	1.16	0.63	1.051	0.307
		Vinylsiloxanether	10	0.85	0.70		
Open tray	Error rate for parallel implants (%)	Polyvinyl siloxane	10	0.46	0.34	0.763	0.455
		Vinylsiloxanether	10	0.35	0.28		
	Error rate for angulated implants (%)	Polyvinyl siloxane	10	0.51	0.30	0.658	0.519
		Vinylsiloxanether	10	0.59	0.27		
Open tray with sandblasting and adhesive coating of impression copings	Error rate for parallel implants (%)	Polyvinyl siloxane	10	0.39	0.22	2.304	0.033*
		Vinylsiloxanether	10	0.19	0.17		
	Error rate for angulated implants (%)	Polyvinyl siloxane	10	0.33	0.27	0.911	0.375
		Vinylsiloxanether	10	0.46	0.34		

## Discussion

Impression is one of the important steps that determine the accuracy of the cast and the precision of fit of the prosthesis. There are various materials and techniques for making an impression. The accuracy of the newly introduced VSE impression material is not well documented and hence, this study was conducted by comparing the accuracy of VSE with PVS.

In the context of this study, the distance between the IA was kept at 10 mm to better represent the distance between two molars. The analogs were placed parallel as well as at 20-degree mesial angulation and the effect of these was evaluated. The three implants were placed at different angulations to better simulate clinical situations that require the placement of angulated implants. A value of 20-degree was used as the angulation in the second-molar region can be between 20 and 25-degree. In this study, PVS and VSE were used in the one-step technique as Wenz and Hertramp reported that the one-step technique is more accurate than the two-step technique [22]. Moreover, the one-step technique is simple to perform and takes less time when compared to the two-step technique and at the same time records fine surface details.

The results of the present study showed that the open tray technique was more accurate than the closed tray technique and is in accordance with the studies conducted by Martínez-Rus et al., Cabral and Guedes, and Wöstmann et al. [23-25]. In regard to implant angulation, the results of the present study showed that for parallel implants, there was no significant difference between the techniques for PVS and is similar to the findings reported by Nakhaei et al. and Osman et al. [26,27]. However, for angulated implants, there was a significant difference between the closed tray technique and the open tray technique which is in accordance with Osman et al. [27]. When implants are placed at different angles, there is increased deformation of impression material upon removal by the closed tray technique. The more the angulation of implants, the more the degree of deformation due to the increased amount of stress generated in the material. However, for the open tray technique, the impression copings remain in the impression and minimize the effect of implant angulation.

The results of the present study showed that open tray with sandblasting and adhesive coating of the impression copings technique demonstrated a higher degree of precision than closed tray and open tray techniques. This is in accordance with Vigolo et al. in which, the master casts obtained with roughened and adhesive-coated impression copings showed a significantly lower amount of rotational movement than the master cast obtained with non-modified impression copings [28,29]. This can be attributed due to the roughening of the external surface of the impression copings by sandblasting and application of an impression adhesive coating on the roughened surface before the final impression procedure leading to better bonding between the impression copings and impression material, thereby counteracting the stresses during removal. This improves the accuracy of the impression and thereby the cast. In clinical situations when the impression is to be made by the open tray technique, this simple and less time-consuming procedure can be considered before impression making in order to improve the accuracy of the impression.

The results of the study showed that there was no significant difference between the two materials for the closed tray technique and similarly for the open tray technique which is similar to the findings of Conrad et al. [10]. However, we observed that for the open tray with sandblasting and adhesive coating of the impression copings technique, VSE exhibited more accuracy than PVS for parallel implants and was statistically significant (P = 0.033).

The improved accuracy of VSE can be attributed due to its hydrophilic nature, good flow, elastic recovery and tear resistance. The hydrophilic nature and good flow resulted in improving wettability and reproduction of fine details. Elastic recovery and tear resistance prevented distortion of the material upon stress generated during removal.

This study has the following limitations. It was an in vitro study in which several factors were under control. In clinical situations, the effect of various factors like saliva and blood can affect the accuracy of impression and the cast and hence further in-vivo studies are needed in order to support the findings of the present study. Moreover, the results of the present study were limited to three implants and may not be relevant for impressions of higher or lower numbers of implants.

## Conclusions

Within the limitations of this in vitro study, we found that the open tray with sandblasting and adhesive coating of the impression copings technique produced the most accurate cast followed by the open tray technique and closed tray technique. For parallel implants, VSE by an open tray with sandblasting and adhesive coating of the impression copings technique produced the most accurate cast. For angulated implants, there was no statistically significant difference between the two materials (PVS and VSE) and the open tray with sandblasting and adhesive coating of the impression copings technique produced the most accurate cast.

## References

[1] Asawa N, Bulbule N, Kakade D, Shah R (2015). Angulated implants: an alternative to bone augmentation and sinus lift procedure: systematic review. J Clin Diagn Res.

[2] Peñarrocha-Oltra D, Candel-Martí E, Ata-Ali J, Peñarrocha-Diago M (2013). Rehabilitation of the atrophic maxilla with tilted implants: review of the literature. J Oral Implantol.

[3] Aparicio C, Perales P, Rangert B (2001). Tilted implants as an alternative to maxillary sinus grafting: a clinical, radiologic, and periotest study. Clin Implant Dent Relat Res.

[4] Sorrentino R, Gherlone EF, Calesini G, Zarone F (2010). Effect of implant angulation, connection length, and impression material on the dimensional accuracy of implant impressions: an in vitro comparative study. Clin Implant Dent Relat Res.

[5] Elshenawy EA, Alam-Eldein AM, Abd Elfatah FA (2018). Cast accuracy obtained from different impression techniques at different implant angulations (in vitro study). Int J Implant Dent.

[6] Selvaraj S, Dorairaj J, Mohan J, Simon P (2016). Comparison of implant cast accuracy of multiple implant impression technique with different splinting materials: an in vitro study. J Indian Prosthodont Soc.

[7] Arora A, Upadhyaya V, Parashar KR, Malik D (2019). Evaluation of the effect of implant angulations and impression techniques on implant cast accuracy - an in vitro study. J Indian Prosthodont Soc.

[8] Buzayan MM, Yunus NB (2014). Passive fit in screw retained multi-unit implant prosthesis understanding and achieving: a review of the literature. J Indian Prosthodont Soc.

[9] Goodacre CJ, Bernal G, Rungcharassaeng K, Kan JY (2003). Clinical complications with implants and implant prostheses. J Prosthet Dent.

[10] Conrad HJ, Pesun IJ, DeLong R, Hodges JS (2007). Accuracy of two impression techniques with angulated implants. J Prosthet Dent.

[11] Shemtov-Yona K, Rittel D (2015). An overview of the mechanical integrity of dental implants. Biomed Res Int.

[12] Hsu YT, Mason SA, Wang HL (2014). Biological implant complications and their management. J Int Acad Periodontol.

[13] Lang NP, Wilson TG, Corbet EF (2000). Biological complications with dental implants: their prevention, diagnosis and treatment. Clin Oral Implants Res.

[14] Wee AG (2000). Comparison of impression materials for direct multi‑implant impressions. J Prosthet Dent.

[15] Ma J, Rubenstein JE (2012). Complete arch implant impression technique. J Prosthet Dent.

[16] Kar S, Tripathi A, Singh J, Ramkumar J (2022). Comparison of dimensional accuracy of elastomeric impression materials using 3D laser scanner. Med J Armed Forces India.

[17] Khan SA, Tushar Tushar, Nezam S, Singh P, Kumari N, Singh SS (2020). Comparison and evaluation of linear dimensional accuracy of three elastomeric impression materials at different time intervals using vision inspection system: an in vitro study. J Int Soc Prev Community Dent.

[18] Berg JC, Johnson GH, Lepe X, Adán-Plaza S (2003). Temperature effects on the rheological properties of current polyether and polysiloxane impression materials during setting. J Prosthet Dent.

[19] Lu H, Nguyen B, Powers JM (2004). Mechanical properties of 3 hydrophilic addition silicone and polyether elastomeric impression materials. J Prosthet Dent.

[20] Apinsathanon P, Bhattarai BP, Suphangul S, Wongsirichat N, Aimjirakul N (2022). Penetration and tensile strength of various impression materials of vinylsiloxanether, polyether, and polyvinylsiloxane impression materials. Eur J Dent.

[21] Re D, De Angelis F, Augusti G, Augusti D, Caputi S, D'Amario M, D'Arcangelo C (2015). Mechanical properties of elastomeric impression materials: an in vitro comparison. Int J Dent.

[22] Wenz HJ, Hertrampf K (2008). Accuracy of impressions and casts using different implant impression techniques in a multi-implant system with an internal hex connection. Int J Oral Maxillofac Implants.

[23] Martínez-Rus F, García C, Santamaría A, Özcan M, Pradíes G (2013). Accuracy of definitive casts using 4 implant-level impression techniques in a scenario of multi-implant system with different implant angulations and subgingival alignment levels. Implant Dent.

[24] Cabral LM, Guedes CG (2007). Comparative analysis of 4 impression techniques for implants. Implant Dent.

[25] Wöstmann B, Rehmann P, Balkenhol M (2008). Influence of impression technique and material on the accuracy of multiple implant impressions. Int J Prosthodont.

[26] Nakhaei M, Madani AS, Moraditalab A, Haghi HR (2015). Three-dimensional accuracy of different impression techniques for dental implants. Dent Res J (Isfahan).

[27] Osman MS, Ziada HM, Abubakr NH, Suliman AM (2019). Implant impression accuracy of parallel and non-parallel implants: a comparative in-vitro analysis of open and closed tray techniques. Int J Implant Dent.

[28] Vigolo P, Majzoub Z, Cordioli G (2000). In vitro comparison of master cast accuracy for single-tooth implant replacement. J Prosthet Dent.

[29] Vigolo P, Majzoub Z, Cordioli G (2003). Evaluation of the accuracy of three techniques used for multiple implant abutment impressions. J Prosthet Dent.

